# A Psychobiographical Study of Intuition in a Writer's Life: Paulo Coelho Revisited

**DOI:** 10.5964/ejop.v13i3.1184

**Published:** 2017-08-31

**Authors:** Claude-Hélène Mayer, David Maree

**Affiliations:** a Institut für Therapeutische Kommunikation und Sprachgebrauch, Europa-Universität Viadrina, Frankfurt (Oder), Germany; b Department of Psychology, University of Pretoria, Pretoria, South Africa; Webster University Geneva, Geneva, Switzerland; The Maria Grzegorzewska University, Warsaw, Poland

**Keywords:** psychobiography, Paulo Coelho, intuition, life study, rationality, writer

## Abstract

Intuition is defined as a form of knowledge which materialises as awareness of thoughts, feelings and physical sensations. It is a key to a deeper understanding and meaningfulness. Intuition, used as a psychological function, supports the transmission and integration of perceptions from unconscious and conscious realms. This study uses a psychobiographical single case study approach to explore intuition across the life span of Paulo Coelho. Methodologically, the study is based on a single case study, using the methodological frame of Dilthey's modern hermeneutics. The author, Paulo Coelho, was chosen as a subject of research, based on the content analysis of first- and third-person perspective documents. Findings show that Paulo Coelho, as one of the most famous and most read contemporary authors in the world, uses his intuitions as a deeper guidance in life, for decision-making and self-development. Intuitive decision-making is described throughout his life and by referring to selected creative works.

"I believe in intuitions and inspirations...

I sometimes FEEL that I am right.

I do not KNOW that I am."

Albert Einstein

During the past few decades, researchers have explored the topics and interconnections of cognition (Frederick, 2005), intuition (Dane & Pratt, 2007) and emotion (Bechara, Damasio, & Damasio, 2000) in the context of decision-making across disciplines (Simon, 1987). The key questions of research are how cognition, intuition and emotions are defined and how they contribute to construct meaningfulness and meaningful decisions in life (Mayer, 2015a).

Psychobiographical research, as the study of the life of "historically significant and extraordinary individuals" (Fouché & van Niekerk, 2010, p. 2) and contribute to explore the topic over their entire life spans with the aim to uncover and reconstruct the lives of individuals psychologically with regard to intuition and intuitive decision-making. Intuition has until now hardly been addressed in psychobiographical research – psych biographical studies often rather use developmental or health-related theories which are then applied to a person's life (Burnell, 2013).

Particularly during the past years, psychobiographies as research method, as well as a theoretical approach have gained international recognition (Alexander, 1990; Fouché & van Niekerk, 2010; van Niekerk, 2007), exploring the lives of outstanding individuals, such as politicians, actors, religious leaders, psychologists, artists and writers (Eliastam, 2011). In psychobiographic studies, a general research gap has been identified to contribute to a new and complex psychological understanding of extraordinary individuals to act as role models (Mayer, 2015b, 2015c). The constructive use of intuition and the positive intuitive decision-making process are aspects of a positive psychology perspective which is according to Luthans (2002) needed in research and according to Mayer (2015c, 2017) needed in psychobiographical works. This is particularly required with regard to the long tradition of pathogen orientations in psychobiographical research which need to be expanded, if not overcome (Kőváry, 2011).

The extraordinary person studied is Paulo Coelho, the world-known writer and novelist who has overcome many difficulties and challenges in his life to become a healthy and faithful person (Mayer, 2015b, 2015c; Mayer & Maree, in press). In this article, it is assumed that this development of a person is based to a large extent on intuition and constructive, positive decision-making processes. Although the writer and his work have been studied extensively (Martin, 2012; Mertel, 2000; Morais, 2009), no psychobiographical work could be found that deals with intuition and intuitive decision-making processes in psychobiographical studies in general or with regard to Paulo Coelho in particular.

Therefore, a research gap has been identified in research on psychobiographical research on Paulo Coelho, and on intuition and intuitive-decision making with regard to qualitative studies across the life span. However, it is assumed that studies across the life span could contribute to new information on how intuition and intuitive decision-making are used and/or developed across a course of a lifetime. The purpose of this study is, therefore, to uncover and reconstruct the selected individual's life through the focus on intuition and intuitive decision-making by exploring conscious and unconscious life decisions. In doing so, this study provides new insights and a new understanding and perspective on the writer Paulo Coelho his life, health, faith and success.

The study aims to explore the uniqueness of Paulo Coelho by focusing in-depth on intuition and intuitive decision-making processes over his life span, with special attention to psychological descriptions of the behaviour, achievements and failures (Elms, 1988) of this extraordinary writer to, for example, provide inspiration for self-reflection (Runyan, 1984) or general psychobiographical interest and the development of the theories applied (Carlson, 1988).

## Psychobiography

Psychobiographies have become a vivid research area in psychology (Kőváry, 2011). They have gained international interest (Fouché, 2015; van Niekerk, 2007) and have also reached beyond disciplinary borders. Psychobiography is defined as "the study of historically significant and extraordinary individuals over their entire life spans with the aim to uncover and reconstruct their lives psychologically" (Fouché & van Niekerk, 2010, p. 2).

Extraordinary individuals, such as politicians, artists and writers, are studied in psychobiographies in single (Eliastam, 2011) or multiple case studies (Schultz, 2005). In these studies, the person becomes the "focus of attention" (Perry, 2012, p. 134). Psychobiographies are anchored in biographical and psychological studies (McAdams, 2006). Often Freud's study on Leonardo Da Vinci is emphasised as a first psychobiography, analysing a life of an outstanding person based on a psychological theory (Kőváry, 2011).

Psychobiography has been criticised by mainstream psychology perspectives as elitist, simplistic, reductionist and holistic (Schultz, 2005; Stroud, 2004), whilst it has been valued for empowering and in-depth visions on single lives and personal histories (Schultz, 2005). This study on Paulo Coelho focuses on the topic of intuition in the writer's life and selected creative accounts. Psychobiographical research on writers has flourished during the past decades and several works have been published (Holm-Hadulla, 2013; Kasser, 2013; Neumann, 2003; Runyan, 2005).

## The Life of Paulo Coelho

This psychobiographical study focuses on the extraordinary life of the Brazilian writer and novelist Paulo Coelho. He was born on 24 August 1947 in Rio de Janeiro, Brazil and was raised by devout Catholic parents, his father being an engineer with a very logical, reasonable and clear vision and worldview (Morais, 2009). During adolescence Coelho was sent to a mental hospital three times by his parents because he did not comply with his parents expectations and due to the fact that he just wanted to become a successful writer at any cost. In his twenties, he was arrested and tortured in Brazil (Encyclopaedia Britannica, 2013), but always kept dreaming his dream of becoming a writer. After his release he started studying law, but dropped out to travel. He changed his life radically at the age of 36 years, after a pilgrimage to Santiago de Compostela in Spain, when he experienced a spiritual awakening and felt inspired to write the book, The Pilgrimage (Coelho, 1987). Only one year later, he wrote The Alchemist (Coelho, 1988) in the course of a two-week spurt of creativity. The Alchemist was Paolo Coelho's break-through as an international author. Since then he has published books at a rate of about one every two years. In 2013, approximately 150 million copies of his books were published in a least 71 languages. Several of his books are autobiographic in nature (Sheme Mary, 2013).

## Intuition

Research has highlighted the questions, how cognition, intuition and emotion interact, relate and influence each other (Hänsel, 2002), particularly in key situation in life. Part of the discourse is the question how intuition influences intra- and inter-personal cognitive understanding and action-related systemic processes (Hänsel, 2002). Research shows that the emotional system plays a mediating role between cognition and behaviour in decision-making processes which refers to the importance of intuition in these processes (De Martino, Kumaran, Seymour, & Dolan, 2006). Intuition and intuitive decision-making have therefore become a relevant part in the psychological discourse on decision-making in important life decisions (Rand & Epstein, 2014).

In early decision-finding process research (Simon, 1987), rational and intuitive decision-making processes have been defined as separated. Only from the 1990's onwards, the interplay of rational (cognitive) and intuitive decision-making processes have become of interest in research (Gigerenzer, 2007; Kahneman, 2011; Kahneman & Frederick, 2002; Kahneman & Klein, 2009; Klein, Moon, & Hoffman, 2006). Several of these discourses touch on the theories about "sense-making" (Klein, Moon, & Hoffman, 2006) and are thereby connected to the question of "how people make sense out of their experience in the world", as highlighted during the 1990's by Duffy (1995). Some of the discursive ideas on intuition and intuitive decision-making have also been associated with the creation of sense-making, meaningfulness and systemic theories, such as system theories, and contexts (Mayer, 2015a; Mayer & Boness, 2013). According to Mayer (2015a), intuition might fulfil the purpose, task and function to create systemic perceptions and base decision-making on a more holistic level, including rationality, emotion, conscious thoughts and unconscious knowledge than just base decision-making on the narrower and more linear concept of rationality only.

Intuition is biologically determined and which is an emotional reaction based on the anticipated consequences of a valued decision-making process (Bechara, Damasio, Tranel, & Damasio, 1997). It is a form of knowledge that expresses itself in thoughts, feelings and physical sensations in connection with a deeper perception and understanding of sense-making (Sadler-Smith & Shefy, 2004, p. 76). Jung (1933, pp. 567-568) emphasises that intuition is a psychological function which transmits perceptions in an unconscious way. This psychological function is connected to terms of "thoughts and preferences that come to mind quickly and prior to rational analysis" (Kahneman, 2011, p. 697). Intuition is, however, also defined as "acts of recognition" (Simon, 1996, p. 89). Therefore, it is understood as a key competence to create comprehensibility, manageability and meaningfulness which are at the same time the key components of a sense of coherence (Mayer, 2015a).

## Intuition as Knowledge System for Decision-Making

Accoding to Bechara, Damasio, Tranel, and Damasio (1997) and Klein (1998) cognition, intuition and emotion and their complex interplay seem to be a complex intra-personal knowledge system and thereby create the basis for decision-making processes in individuals. Based on research in real-world scenarios, individuals use various conscious and unconscious abilities, skills, perceptions and sources of energy to make decisions (Klein & Klinger, 1991). It has been pointed out that deductive and logical thinking processes or the analysis of statistics and probabilities are not practical or relevant to make useful decisions (Klein, 1998, p. 3), such as the slow processes. Klein (1998) highlights that in real-world scenarios, decision-making methods need to be practical and based on holistic perceptions which draw in as many data and information as possible, including consciously and unconsciously perceived systemic knowledge which is based on intuition, mental stimulation in terms of mental activity, metaphoric knowledge, narrations and feelings. Thereby, intuition and expertise are not to be understood as contraries and conflictual (Kahneman & Klein, 2009), but rather as integrative: intuitive decision-making is based on a qualitative and valuable judgement of the natural environment. It is anchored in a distinct assessment and analysis of the real world situation which is associated with a holistic experience and perception of the situation. Hogarth (2001) emphasises that this holistic experience and perception is improved with each and every experience and intuitive decision-making process. Through the repeated use of intuition and intuitive decision-making processes, adequate intuitive decisions can be made even in extraordinary and irregular situations, based on the access of holistic experiences and perceptions. However, the subjective experiences are not necessarily an indicator of precise decision-making (Kahneman & Klein, 2009). An intuitive synthesis is usually based on three complex aspects: the judgement of a situation, the previous experiences and the manifestation in a "gut feeling" (Khatri & Ng, 2000, p. 67). Intuition therefore involves cognitive processes (and is not contrary to them) and emotions (which are not the only source of intuition). Very strong emotions towards an experience or situation, however, might interfere with an adequate intuitive operation and perception (Ray & Myers, 1990) and it might occur that strong emotions interfere with transferring valuable intuitive messages into the consciousness and thereby into conscious intuitive decisions (Vaughan, 1990).

During the past decade, Evans (2007) highlights that the "dual model" of decision-making is strongly relevant: in System 1 operations, decisions seem to be automatic, non-voluntarily and without effort. In System 2 operations – which are usually used during self-reflection or in conscious mathematical problem-solving scenarios – consciously decisions and actions are relevant and are based on effort and conscious rationality (Kahneman & Klein, 2009, p. 519). System 1 and System 2 operations are two parallel existing knowledge systems which are interacting with each other and which are described as "knowing (intuition-as-expertise) and sensing (intuition-as-feeling)" (Sadler-Smith & Shefy, 2004, p. 76). Also Kahneman (2011) emphasises that there is a dichotomy between two modes of thought, referring to the System 1 (fast, instinctive, automatic, frequent, stereotypic, subconscious and emotional) and the System 2 (slow, deliberative, effortful, infrequent, calculating, conscious and logical). These two knowledge systems combine rationality and intuition within decision-making and contribute to a more holistic understanding, interpretation and managing of the world and the decisions to be made.

## Research Methodology

This research study uses a psychobiographical research methodology. It is based on a research design based on a person-centered single case (Elms, 2007; Yin, 2009) within the frame of Dilthey's modern hermeneutics (Dilthey, 2002). The study uses a holistic approach of the individual to explore the concept of intuition throughout the life of the writer. Through the hermeneutical approach, "Verstehen" (understanding) is created (Dilthey, 2002) by applying a self-reflective attitude of the researcher towards the exploration and interpretation of the text of lived experiences (Ratner, 2002) in the life of Paulo Coelho. Dilthey’s understanding is defined as an appropriate and suitable approach to this study, due to the fact that it integrates the main aim of interpreting ideas, purposes and other mental states expressed in reality (Babbie & Mouton, 2006; Dilthey, 1976). Dilthey`s (2002) hermeneutic tradition was chosen due to his strong emphasise on biography on the one hand and on self-reflection in hermeneutics (in comparison to Heidegger, Gadamer, Ricoeur or Levinas) on the other hand. His approach was chosen due to his pioneering work in the Humanities which particularly focused on the uniqueness and wholeness of the individual whilst referring to the analysis and descriptiveness in Psychology (Kőváry, 2011).

### Sampling

Paulo Coelho was purposefully chosen as a subject of research. This purposeful sampling, which is defined as being based on a variety of sampling criteria including the specialist knowledge of the research issue (Oliver, 2006), includes the personal interest in the life and creative works of the writer by the authors, the importance of intuition in the creative works and the life of the writer and the person Paulo Coelho, as an outstanding author.

### Data Collection and Analysis

Data was collected through first- and third-person documents (Allport, 1961). These documents^i^ included autobiographical accounts and selected literary products of the author (altogether 33), twelve Internet sources, journal and newspaper articles, three interviews (two online and one in print; Arias, 2001), and one video documentary (first-person documents; Binding & Mirow, 2010), Coelho’s biography (Morais, 2009), one interview (Arias, 2001), and two case studies on selected creative works (e.g., Mihály, 2012) were used for the analysis.

Data were analysed through content analysis based on the five-step process of Terre Blanche, Durrheim, and Kelly (2006, pp. 322-326): (a) familiarisation and immersion; (b) inducing themes; (c) coding; (d) elaboration; and (e) interpretation and checking. The content analysis led to the definition of themes, categories and codes (Yin, 2009). In the beginning of the analysis the researchers identified several life themes, such as health, faith, spirituality, creativity and intuition which were identified throughout the writer's life. They then coded the first and third data according to the theories applied and induced codes using inductive and deductive processes of analysis. As organising criteria, the researchers used the different life stages, reaching from birth to the end of the sixties, thereby emphasising incidents which occurred in the context of intuition throughout the life of Paulo Coelho.

Data are presented in a qualitative form of a narrative – highlighting outstanding incidences –, reporting the findings on intuition along the writer's life's stages. The authors defined the life stages as an organising principle of reporting the findings, as often done in qualitative psychobiographies (e.g., Eliastam, 2011; Perry, 2012). The authors further decided on a combined data presentation, integrating analysis, findings and discussion and thereby integrating descriptions of the findings and their interpretation. This way of presenting the findings, results in establishing the qualitative research criteria mentioned below and the creation of meaning, whilst presenting the findings (Sandelowski, 2010).

### Qualitative Research Criteria

To ensure qualitative research quality, the researchers applied qualitative research criteria (Yin, 2009), particularly referring to trustworthiness, credibility, dependability, transferability and confirmability (Sinkovics, Penz, & Ghauri, 2008). Further on, we applied the triangulation of theories, research methods and data (Casey & Murphy, 2009).

### Ethical Considerations

Ethical clearance was provided by the University of Pretoria in Pretoria, South Africa. Research ethics included particularly the respectful treatment of intimidate details of the writer (Elms, 1994) and non-maleficence (Schurink, 1998). Research ethics are valued as strongly important in this psychobiographical study due to the fact that the subject of research is alive and not deceased yet (Elms, 1994).

## Findings

### Intuition During Paulo Coelho's Childhood (1947-1959)

Paulo Coelho de Souza was born on 24 August 1947 to Lygia Araripe Coelho de Souza and Pedro Queima Coelho de Souza. He experienced a complicated birth in Rio de Janeiro, Brazil, that he hardly survived (Morais, 2009). Pedro, his father, was a logical and rational person who worked as an engineer. He had a linear thought-style and a clearly defined vision, whilst Lydia, the mother, was focused on a strong Christian, Catholic belief (Coelho, 2006, p. 11).

Paulo's life started with an act of faith and prayer by his mother, Lydia, who intuitively started praying to Saint Joseph when she realised that her son was struggling to survive his birth (Morais, 2009). She understood that her son would not survive – which was a key situation in her personal life – and began to connect her cognitive understanding of the situation with the intuitive wish to act (as described in Hänsel, 2002). Her emotions thereby played a mediating role between cognition and behaviour whilst making the decision to pray (as described in De Martino, Kumaran, Seymour, & Dolan, 2006).

Surprisingly, Paulo survived the challenges of his birth and in an act of "sensemaking" (Klein, Moon, & Hoffman, 2006) responded to the question posed by Duffy (1995, p. 6) "how people make sense out of their experience in the world". She then dedicated Paulo's life to St Joseph (Morais, 2009). Based on Paulo's introduction to life, childhood was strongly influenced by defining and living his relationship with God through prayers (Morais, 2009).

At the age of six, Paulo started school in the context of a strict Catholic-based primary school system which enforced the values of faith, respect and hard work (Morais, 2009). However, Paulo intuitively new that he was born to read and to write books and started to focus on reading books extensively from the age of eight years, whilst loosing interest in his work at school. Already at this time of his life, Paulo was aware of his emotional pull towards reading and towards books which can be interpreted as an intuition as a psychological function according to Jung (1933) who defined this psychological function as connected to thoughts and preferences that come to mind quickly and prior to rational analysis (Kahneman, 2011). However, these unconscious preferences might be viewed as the first "act of recognition" (intuition), as defined by Simon (1996, p. 89). This first "act of recognition" on Paulo's way of becoming a world known writer, must be seen as being based on his probably conscious decision to prefer reading, stories and books to school.

### Integrating Intuitive and Rational Decisions Towards Becoming a Writer During Adolescence (1959-1966)

Six years later, Paulo started writing a diary. He "knew" that he only wanted to become a writer and developed the idée fixe of becoming one (Morais, 2009, p. 62). At this point in time, Coelho combined and integrated a rational and cognitive and conscious decision-making process with his intuitive and emotional (Kahneman & Klein, 2009) and up to this point in time rather unconscious idea from his childhood to spend most of the time reading which seems in retrospect to having been an unconscious, but intuitive decision for preparation for his own career in writing as he highlighted later in his biography (Morais, 2009; as described in Klein & Klinger, 1991). Coelho attended a school with a strict Jesuit tradition which he did not enjoy (Morais, 2009), struggled with his belief in God, feelings of guilt, the interest in religion on the one hand and the desire to break free from his strict educational and familial background and experienced a deep crisis when he lost his grandfather (Arias, 2001). He made new friends who included him into the youth scene of artists, left-wingers and the world of theatre. He thereby entered a new world which was a strong alternative model to his familiar upbringing. Morais (2009) describes that Coelho, as almost by accident, met his new friends that introduced him to this new, fascinating world. He did not consciously connect to this scene, but found himself in midst the scene, getting inspired by the artistic worldviews of the fellows. His first introduction to the artistic world is described as happening coincidentally (Morais, 2009), almost as the application of System 1 operations which happen "automatically" and without effort (Evans, 2007, p. 5).

However, being torn apart between his parents desire to make him a successful engineer and his personal dream to become a writer, Coelho experienced strong emotional instability (Morais, 2009). His parents admitted him to a mental hospital thrice between 1965 and 1967 (Jeffries, 2013) which impacted strongly on his doubts towards life, the implementation of his dream to become a writer and his self-doubts (Arias, 2001; Morais, 2009).

### The Rationality of the Belief in the Devil and the Intuitive Return to Christianity in the Twenties (1967-1976)

In the next life decade, Paulo consumed drugs and entered a new world of personal freedom, away from his parents. He left university and broke off with his studies in law and engaged more and more in theatre play productions whilst he continued writing (Morais, 2009). Through the rational decision to stop studying a subject he was not interested in, Coelho served intuitively his dream to become a successful and famous writer. Intuitively, Coelho knew – as described in Sadler-Smith and Shefy (2004) through his intuition as expertise that he would not be happy to become a lawyer, whilst he could feel strongly on the level of sensing and "intuition-as-feeling" (Sadler-Smith & Shefy, 2004, p. 81) that his world was the world of the artists, actors and writers. His quick decision to leave the university was thereby based on his "gut feeling" (Khatri & Ng, 2000, p. 67), as well as on his rational decision that he did not want to become anything else than a writer. During his middle twenties, Coelho was often depressed and frustrated that he had not started to become a famous writer (Morais, 2009) and therefore made the conscious and rational decision to turn away from his Christian background completely and to believe in the devil and the occult (Arias, 2001). He believed he was "a magician preparing for his dawn" (Morais, 2009, p. 181) and made a contract with the devil: he sold the devil his soul and expected in return the dark forces to support him in reaching his aim to become a famous writer (Morais, 2009). Coelho consciously made the pact with the devil, following his strong inner desire to excel. He thereby applied a new view of the world, good and evil and used a new cognitive understanding of the world to implement new action-related systemic processes (as described in Hänsel, 2002): he used his new belief system consciously to promote himself as a writer and moved away from his more intuitive knowledge and belief in God and the world. Coelho thereby turned his frustration that he had experienced of his failure to become a famous writer into a new occult and rather rational and strategic approach to reach his aim and thereby he made sense out of his negative experiences (as discussed in Duffy, 1995; Klein, Moon, & Hoffman, 2006) whilst knowing intuitively, particularly through the intuition-as-feeling throughout the decade that he was striving towards the right aim and his personal dream to become a writer. His new belief therefore became a meaningful way of dealing with his frustrations of his stagnations as a writer and represents a way of trying to manage his inability to proceed with his career by himself (as described in Mayer, 2015a; Mayer & Boness, 2013).

Besides his involvement in constructing a new belief system and active magical influence to fulfill his dream, Paulo married Gisa, his first wife, met Raul Seixas who became his friend and business partner and got into music productions whilst practicing magic to boost his success (Morais, 2009).

Because of his frustrations of being unsuccessful, Coelho defined himself as the "Prince of Darkness", sold his soul to the devil to make his dream come true and dissolved the contract one hour later and turned away from Satanism completely after having been introduced to the first initiation tire of the sect he had joined which he related to as the phenomenon of the black cloud (Arias, 2001). He panicked and turned back to his childhood belief: Christianity (Morais, 2009). His rational decision to sell his soul to his devil was revised by Coelho by his intuition-as-feeling that he was not doing a right thing. He also decided intuitively to return to Christianity after he experienced fear and after he had realised his wrong and limited decision to become a Satanist through a more holistic understanding, through the recognition of the anticipated consequences of the decision-making process (Bechara, Damasio, Tranel, & Damasio, 1997). Through the initiation experience, Coelho developed a deeper understanding and perception of his sense in the world (as described in Sadler-Smith & Shefy, 2004). He realised immediately, without effort (Evans, 2007) and through "thoughts and preferences that come to mind quickly and prior to rational analysis" (Kahneman, 2011, p. 697) that it was the right point in time to return to Christianity to realise his dream and live his life. He underlined the correctness of his decision by using his "knowing-as-expertise" (Sadler-Smith & Shefy, 2004, p. 76) and Coelho dedicated his life once more to St Joseph (Coelho, 2006), whilst integration a rational approach to celebrate his life and by intuitively knowing holistically that this was his right way to go

### The Intuitive Spurt After the Walk – The Thirties (1977-1986)

After Coelho had divorced Gisa, he had married his second wife, but got divorced quickly again during his twenties. It was only during his thirties that Coelho met his life partner Christina. He knew immediately that she was the woman he had waited for (Arias, 2001) by applying his intuition-as-feeling (Sadler-Smith & Shefy, 2004) and his System 1 operation (Kahneman & Klein, 2009). They travelled through Europe and Coelho experienced his calling to become a writer while listening to church bells tolling in the concentration camp in Dachau and he realised once again that his time as a writer was to come (Arias, 2001, p. 141).

It was during the thirties that Coelho made promised to different Saints (Nhá Chica; Jesus of Prague, St. Theresa) to honour them and return to them when they helped him to become a famous writer (Arias, 2001; Morais, 2009). Coelho thereby combined his rational understanding that he needed help to move towards realising his dream whilst following his intuition stepwise to walk on into this chosen direction (Sadler-Smith & Shefy, 2004).

During one of Christina and Paulo's trips to Europe, he was invited by a member of the catholic order of RAM ("Regnus Agnus Mundi") to join the order as a disciple of a man called J. (Arias, 2001). Paulo had one night to decide and was challenged by this decision. Morais (2009) and Arias (2001) both describe that this decision was a difficult one for Coelho who was term apart by his rational and intuitive decision-making processes (as described in Evans, 2007). He finally decide for the order, based on his "gut feeling", the judgement of the strange situation and offer and his previous experience within the satanic sect (Khatri & Ng, 2000)

With his decision for RAM, Paulo had to follow his Master's advice in many respects and was sent on a pilgrimage to Santiago de Compostela in Spain after he had failed to gain his sword to become a Master of RAM, too (Morais, 2009). After this pilgrimage and a several month long depression, Coelho wrote his first world bestseller (Coelho, 2003): "the pilgrimage" in a two weeks spurt of creativity. It might be assumed that Coelho connected during this journey deeply to his intuitive knowledge as expertise and as feeling and a deeper and even more holistic experience and perception (Hogarth, 2001). However, Coelho got into a depression due to his failure to immediately apply his knowledge and then managed to access his knowledge whilst applying his expertise, his feeling and his rationality towards the process of writing (Kahneman & Klein, 2009). In the two weeks act of creativity, Coelho found himself in "acts of recognition" (Simon, 1996, p. 89) and wrote his bestseller, describing himself as a magus: "Yes, I am a magus, but so is everyone who knows how to read the hidden language of things in pursuit of their personal destiny" (Arias, 2001, p. xix). He had found an effective way to approach and apply his intuition as expertise and knowledge.

### An Integrative Approach to Rationality and Intuition – The Forties (1987-1996)

Paulo’s fourth life decade was characterized by his establishment as a writer and his spiritual development (Coelho, 2003). During this decade, Coelho combined his rational knowledge, his logical thought processes, statistical and management knowledge to manage his success and to expand his celebrity status to make useful decisions on the one hand (Klein, 1998) and his systemic knowledge of networking, mental stimulation, metaphoric knowledge, narrative approaches to life and life narration and access to feelings on the other hand (Klein, 1998). Coelho's success might even be mainly based on his personal competence to combine System 1 operations and System 2 operations during his life and with regard to writing his books and marketing them. As described by Kahneman and Klein (2009), Coelho became during his forties not only a master in RAM, but also a master in integrating intuition and expertise, rationality and intuition whenever needed. He analysed the market for his narrations on the one hand (Arias, 2001; Morais, 2009) and accessed a deeply intuitive knowledge of spirituality on the other hand (e.g., Coelho 2011). The combination of rational thought and intuitive spirituality as a content of his books and as a strategy in the real world to market his book shows that Coelho does not see the differentiation of rationality and intuition as contradictive or conflictual, but that he is able to integrate and combine these two systems in a meaningful and applicable manner. This potential and knowledge to combine rationality and intuition is not only part of Coelho's books written in the forties, such as "The alchemist" (Coelho, 2002), "the Valkryries" (Coelho, 1992), "Brida" (Coelho, 1990), but also in the way how Coelho developed his marketing strategies, his holistic approach to write and sell his books, his life as a writer and his life as a disciple and master of RAM, his business and political contacts and networks, as well as his meditative and contemplative practices.

During the forties, Coelho travelled also to the Road to Rome and developed a feeling and expertise to judge his subjective experiences as either important and/or unimportant for his work as a writer (as emphasised in Kahneman & Klein, 2009; Khatri & Ng, 2000). Situational experiences and/or new relationships were perceived as intuitively important and valuable intuitive messages were transferred into valuable intuitive messages that were then consciously used in selected narrations, scenes and/or descriptions in his books, thereby connecting the visible and the invisible worlds (Coelho, 1990).

### Combining Resources for Success in Life and Creative Works – The Fifties (1997-2006)

In the fifties, Coelho decided intuitively that he was now able to review his experiences made in the youth in the mental hospital and wrote the book "Veronica decides to die" (Coelho, 1998). The book deals with the “acts of recognition” (Simon, 1996, p. 89) that a rational approach to life only and rational decisions might not always lead to the appropriate decisions which are the best for the person.

During the fifties, Coelho wrote several more books in which he explored belief systems, values and norms on a deeper level (e.g., Coelho, 2007) and in which Coelho discussed the question of how important the combination of values and intuition is in comparison to a rational approach to life and sensemaking. In several books, Coelho describes the importance of recognising the complex interplay of cognition, intuition and emotion (Bechara et al., 1997; Klein, 1998) and a complex holistic understanding of the world (Klein, 1998), based on intuition-as-expertise and intuition-as-feeling (Sadler-Smith & Shefy, 2004) and backed-up by a combination of System 1 and System 2 operations (Evans, 2007). In "Veronica decides to die" (Coelho, 1998), for example, Coelho describes the struggle of the protagonist – which Coelho explains is his personal representative (Arias, 2001) – who knows intuitively by feeling what is good for her, but acts against her intuition by making rationally based decisions (i.e., not living her dream, following routines she does not like, holding back her emotions instead of showing them). In the end of the book, the protagonist manages to follow her intuition more wisely whilst taking care of herself through rationally integrated decisions and thereby experiences a personal growth.

In his real life, Coelho applied even more improved the combination of rational and intuitive power to become more successful: he applied for a seat in the Brazilian Academy of Letters, a renowned Brazilian writers' society by applying rational decisions, such as the application as such, and by applying intuitive decision-making by moving to France and waiting patiently for the reaction of the society without interfering more in the process, exploring his own intuition through using the I-Ching (Morais, 2009).

During the fifth decade in Coelho's life, the writer aimed at applying holistic knowledge and combining deep and reflective life knowledge on System 1 and System 2 levels in his life, as well as in his books. It might be assumed that this combination of describing the struggle, the conflictual relationship between rationality and intuition as well as attempt to apply rationality and intuition in his life as well as in the characters in his narration is part of his overwhelming success. Whilst Coelho accepts both systems as interpretations of the world and whilst he applies both systems 1 and 2 (Kahneman, 2011), he repeatedly shows in his narrations, as well as with regard to his life story and the fulfilment to become a famous and successful writer that intuitive approaches and practices are often the ones that a person should follow to reach his/her aims, whilst the rational approaches to life do often limit a person's perceptions, abilities and actions. In his narrations, Coelho pleads for a careful and mindfully applied combination of System 1 and System 2 decisions which might be addressed in affirmations, such as "follow your dream in a mindful way".

### The World Beyond – The Sixties (2007-2015)

The sixth decade of Coelho's life started with a quite birthday in France and was followed by a huge birthday party on St. Joseph's day in Spain (Morais, 2009). This decade of Coelho's life is characterised by more book publications (Coelho, 2008) a documentary on Coelho (Binding & Mirow, 2010), and the bestseller "Aleph" (Coelho, 2011). Particularly the creative work "Aleph" (Binding & Mirow, 2010) refers again to a combination of Coelho's ability to combine rational thought and strategic management of his career in the context of negotiations with his publisher and the application of intuition in the development of the relationship with a young woman on the train. Again, Coelho plays with the System 1 and 2 by describing his thoughts, his dilemmas and his fulfilment of spiritual development in the context of this new relationship by exploring intuition and worlds beyond a rational reality (Binding & Mirow, 2010).

Several of Coelho’s books deal with socio-political criticism during this life's decade. However, "Aleph" is one of the most personal accounts of Coelho's struggle between the two systems 1 and 2 (Kahneman, 2011) and he is asking himself what source can we actually trust? Which knowledge do we trust, what are the "real intentions" in life, what is the desired development? Coelho reflects on holistic perceptions, on values and the deeper levels of existence, purpose in life and which decisions impact how on the individual, his/her life purpose and development? (Coelho, 2011).

Coelho seems to use the decade of the sixties to get into deeper levels of exploring life and lives beyond life by applying his knowledge-as-expertise, his knowledge-as-feeling and trying to resolve the purpose of life and its meaningfulness on a deeper level of reflection and feeling, consciousness and unconsciousness in a holistic way.

## Discussion

### Limitations of the Study

The study is subject to theoretical (intuition) and methodological limitations (Schultz, 2005), as well as to the researcher's bias (Nortjé, Fouché, & Gogo, 2013), the defined qualitative quality criteria (Mayer, 2015c) and the general criticism that this study falls into the category of elitist research which focuses on one single famous, prominent celebrity.

### Conclusion and Recommendations

The focus of this study was the analysis and interpretation of intuition in the life of Paulo Coelho. The life was reconstructed psychobiographically (Fouché & van Niekerk, 2010) to review the life of Paulo Coelho and his creative works in the context of intuition and intuitive decision-making.

The study shows that the career of becoming a successful and international writer did not only start for Coelho with the pilgrimage through France and Spain in 1987 to Santiago de Compostela or the realisation of the "Call to become a writer" in the concentration camp in Dachau, Germany, but already at the age of eight years when Paulo Coelho preferred reading, stories and books to his school work through the intuitive "act of recognition".

The reconstruction of the life of Paulo Coelho refers to the question of the role of intuition and rational and intuitive decision-making in the life of Paulo Coelho, as well as in his creative thoughts. By responding to this question, the study provides new in-depth insights and understanding to the impact of intuition in the life and works of this outstanding writer.

**Figure 1:**
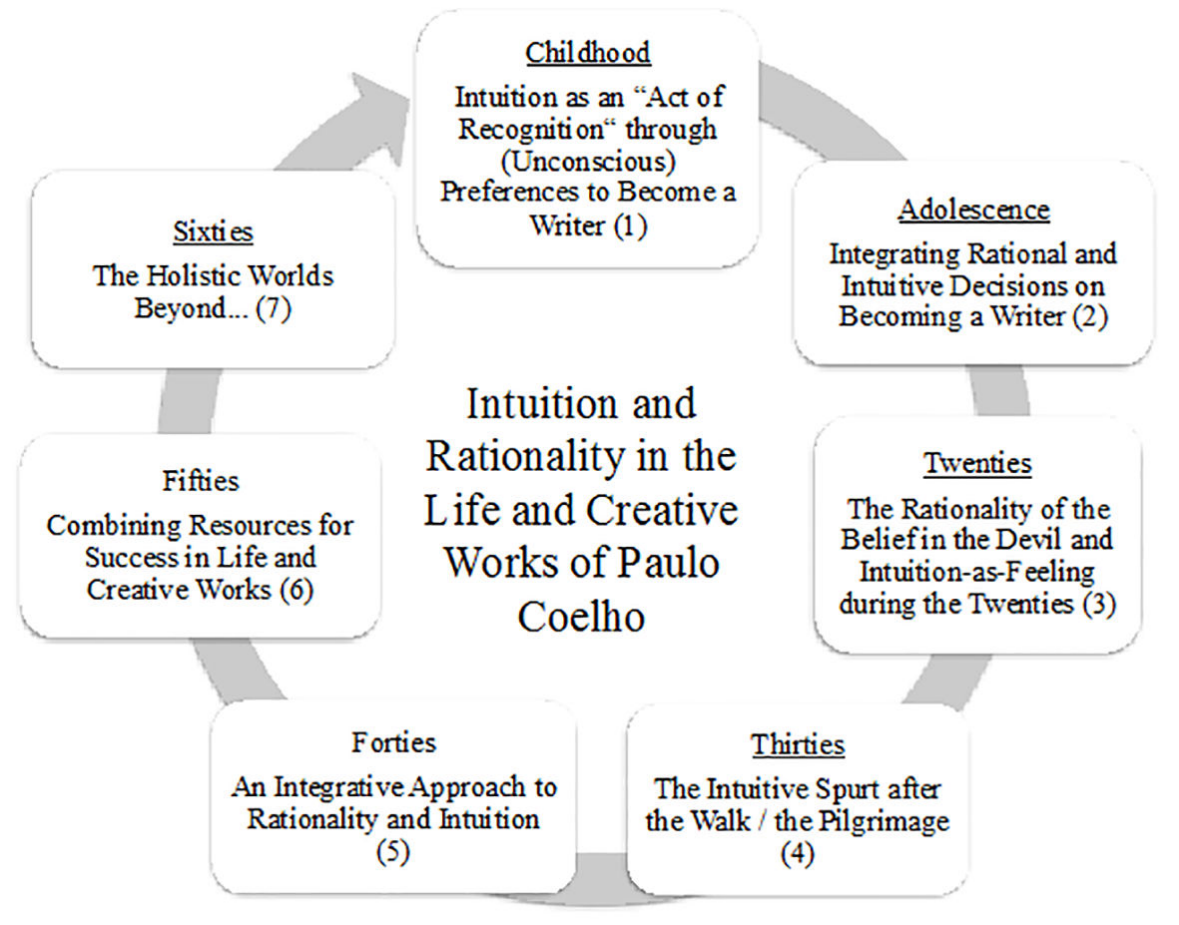
Intuition and rationality across the life span.

Focusing on the life of Paulo Coelho, the study shows that the focus cannot be on intuition only, but also needs to integrate rationality as an important aspect of decision making throughout the life span (see Figure 1).

This psychobiographical study emphasises that intuitive decision-making process to become a writer is already expressed unconsciously and intuitively by Paulo Coelho at the age of eight years and not, as highlighted before during adolescence. The intuitive preparation of Coelho during childhood might play a very important and outstanding role as a marker for his success later in life. The strong urge of reading in Coelho's childhood is therefore interpreted as an early intuitive decision towards the profession of becoming a writer.

During adolescence, Coelho realises consciously his idée fixe to become a writer and thereby starts exploring and integrating rational and intuitive decisions to expand his writing abilities and experiences in artistic, theatre and writing competition contexts.

In the following decade, Coelho loses contact to his intuitive system and decides consciously for a pact with the devil and Satanism to reach his aim of becoming a successful and famous writer. However, through a fearful and outstanding initiation ritual into a Satanistic sect, he bethinks his inner values and returns to Christianity and his childhood belief. Thereby, Coelho, re-accesses his intuitive knowledge and returns onto a path of intuition.

Following this inner revolution, Coelho expresses his intuitive knowledge of expertise and feeling through a spurt of intuition and creativity after having joined RAM and walked the pilgrimage to Santiago.

It happens in the forties that Coelho starts to integrate a balanced approach to rationality and intuition during life, but also in his creative works and narrations. Coelho starts exploring intuitively his spirituality which he shares with the readership whilst aiming to balance rationality with intuition to become spiritually fulfilled, successful and famous.

During the fifties, Coelho explores on a deeper level to combine intuitive and rational thinking through integrating different values in his life and creative works and by expressing his struggles to find out what his actual path looks like – is it the one his rationality opens to him or is it the one that his intuition provides for him?

It is only during the sixties that Coelho finds a way of combining rational and intuitive thoughts and decisions within his narration – particularly in the creative work Aleph (2011) – on faith for himself, on resolving life's riddles based on combining rational and intuitive approaches to meaningfulness.

The findings of this study lead to some theoretical and practical implications, referring to the idea that more long-term studies on intuition and the application of intuitive and rational thinking is needed. Psychobiographies could provide new and important information how intuitive decision-making develops across the life span and the ways in which System 1 and System 2 operate might be consciously explored and integrated to come to holistic and meaningful decisions throughout the life span.

On a practical level, this psychobiographical study might provide new insights into the development, process and expression of intuition and intuitive decision making throughout the life span of a single person. It provides the reader with new ideas on how to balance System 1 and System 2 decisions and might create consciousness around the topic of intuition and the importance of in-depth self-reflection and conscious exploration of the two decision-influencing systems.
